# Compact Spectral Encoding Microscopy by Terrace Grating Optics

**DOI:** 10.1021/acsphotonics.5c02701

**Published:** 2026-02-16

**Authors:** Ori Refael Cohen, Reut Orange Kedem, Leonid Leites, Amit Parizat, Jonathan Jeffet, Lior Laufer, Shay Stern, Yuval Ebenstein, Yoav Shechtman

**Affiliations:** † Russel Berrie Nanotechnology Institute, Technion−Israel Institute of Technology, Haifa 3200003, Israel; ‡ Faculty of Biomedical Engineering, 26747 Technion−Israel Institute of Technology, Haifa 3200003, Israel; § Raymond and Beverly Sackler Faculty of Exact Sciences, 26745 Tel Aviv University, Tel Aviv 6997801, Israel; ∥ Faculty of Biology, Technion−Israel Institute of Technology, Haifa 3200003, Israel; ⊥ Faculty of Electrical and Computer Engineering, Technion−Israel Institute of Technology, Haifa 3200003, Israel

**Keywords:** Optical Microscopy, Point spread function Engineering, Diffraction Gratings, 3D imaging, Diffractive optical element

## Abstract

Spectral information is essential in microscopy, yet many multispectral imaging solutions require increased optical complexity or restrict the spectrum to a few discrete bands. In this work we introduce the Terrace grating optics family: flat, 3D-printable elements that provide continuous spectral encoding, as a plug-and-play add-on component to standard microscopes. The Terrace design preserves the illumination path in widefield operation and integrates trivially with other microscopy phase masks. We introduce two variants: a Single-order Terrace that concentrates energy into one diffraction order for high signal-to-noise ratio, and a Dual-order Terrace that adds a wavelength-independent reference spot, enabling robust color decoding. Using a Fourier-optics model, we show that the wavelength displacement is linearly controlled by the Terrace step-width and refractive-index mismatch. We validate the approach in two settings: snapshot decoding of four-color mRNA barcodes and multicolor NeuroPAL worm imaging with a single camera exposure and instantaneous color readout. Furthermore, we demonstrate continuous spectral and depth encoding masks yielding simultaneous depth and color readout via PSF shape, which remains compact and efficient. Terrace gratings thus offer a practical alternative to prisms and blazed gratings, enabling continuous multispectral encoding and straightforward integration with conventional microscopes.

## Graphical abstract

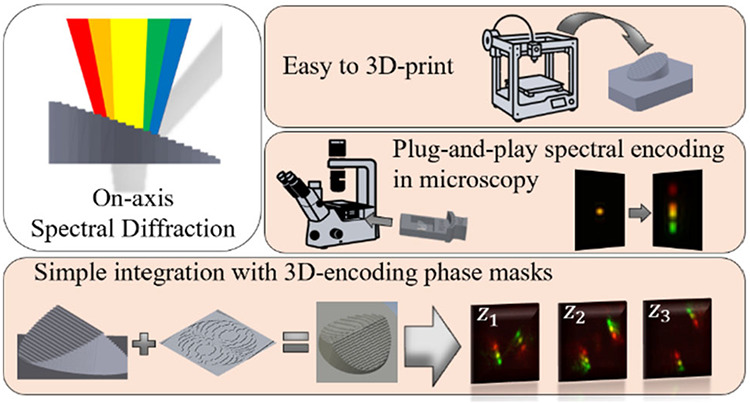

## Introduction

Spectral information is valuable in microscopy, providing insight about sample composition, labeling, functional state, and more. Common approaches for multispectral imaging in microscopy include sequential acquisition using multiple filter sets,^1,2^ simultaneous acquisition with multiple cameras combined with channel-splitting optics,^3^ spectral splitting of the field of view^4,5^ or extension of the optical system.^6^ Spectral information can also be obtained by using point-spread-function (PSF) engineering, on a single optical channel, for a discrete set of wavelengths,^7–9^ which involves a spatial light modulator or a diffractive optical element (DOE) in the Fourier plane. These solutions necessitate additional components for channel splitting, decreased throughput for sequential imaging, compromising the field of view, or mechanical alignment and calibration, and require some degree of expertise.

A widely used element for spectral encoding is the diffraction grating, which can be transmissive or reflective. In their conventional square waveform, gratings diffract light into multiple orders, reducing efficiency and increasing information density in the image plane. One way to improve spectral encoding efficiency is to use a blazed grating, which concentrates most of the diffracted energy into a single order, resulting in a shift by wavelength. This makes blazed gratings attractive for spectral encoding. However, such gratings require highly precise fabrication and are challenging to integrate with additional optical capabilities.

Seeking a method for simple, customizable and extendable integration of spectral splitting in microscopy, we were inspired by Delort et al.,^10^ who presented a structure consisting of rectangular bricks with partial vertical overlap, achieving spectral splitting, and by Yang et al.,^11^ who used a step-like structure for beam steering and beam splitting.

Here, we introduce and demonstrate a continuous spectral encoding component, the Terrace grating, with functionality similar to a transmissive blazed grating but featuring a simple, flat geometry that allows easy handling, low-cost fabrication, as well as straightforward integration with other phase masks, e.g., for 3D encoding, that allows unconventional designs. We derive analytical expressions for Terrace grating design, and using microscale 3D printing and near-index matching,^9^ we fabricate “plug-and-play” spectral encoding phase masks that can be plugged directly into an existing slot downstream of the objective in a conventional inverted microscope. In contrast to conventional blazed gratings with a sawtooth profile, the flat Terrace geometry introduces no angular steering at the mask plane. Consequently, in fluorescence microscopy, illuminated by a beam constituting a small spot at the mask plane (near the back focal plane), the Terrace grating preserves the excitation angle and keeps the collected emission on-axis, so neither the illumination nor the imaging channel is perturbed by beam deflection. Furthermore, the simple geometry is highly compatible with 3D printing fabrication methods and does not require quantization of the inclined blaze angle.

We demonstrate three examples out of a large set of possible designs, tailored for different applications, including single-molecule mRNA imaging, neuronal imaging in*Caenorhabditis elegans* nematodes, and integration of continuous spectral information into 3D encoding phase masks.

## Theory

### Imaging Model

In the scalar approximation, the complex amplitude transmittance of a phase mask *t*(*x*,*y*) is given by

1 $$t\left(x,y\right)=e^{i\phi \left(x,y\right)}$$

where ϕ­(*x*,*y*) is the phase map contributed by the phase mask to the field.^12^ For a Terrace grating composed of two layers with refractive indices *n*_1_ and *n*_2_, respectively, refractive index mismatch Δ*n* = *n*_2_ – *n*_1_, step width *a*, step height *h*, (Figure 1a) and *N* number of steps, the transmittance function can be written as

2 $$t\left(x,y\right)=\sum_{m=-\left(\left(N-1\right)/2\right)}^{\left(N-1\right)/2}e^{i2\pi \Delta n/\lambda \cdot \mathrm{mh}}\cdot rect\left(\frac{x-\mathrm{ma}}{a}\right)$$

whereas rect­(*x*) denotes the rectangle function along the *x*-axis

3 $$rect\left(\frac{x}{a}\right)=\{\begin{array}{l} 1\\ 0 \end{array},\begin{array}{l} \left|x\right|<\frac{a}{2}\\ \mathrm{otherwise} \end{array}$$

When the phase mask is placed at the back focal plane (BFP) of the objective, the complex field in the image plane is proportional to the Fourier transform of the field at the BFP

4 $$U_{i}\left(x_{i},y_{i}|x_{o},y_{o}\right)\propto F\left\{U_{\mathrm{bfp}}\left(x_{\mathrm{bfp}},y_{\mathrm{bfp}}|x_{o},y_{o}\right)\right\}$$

**Figure 1:**
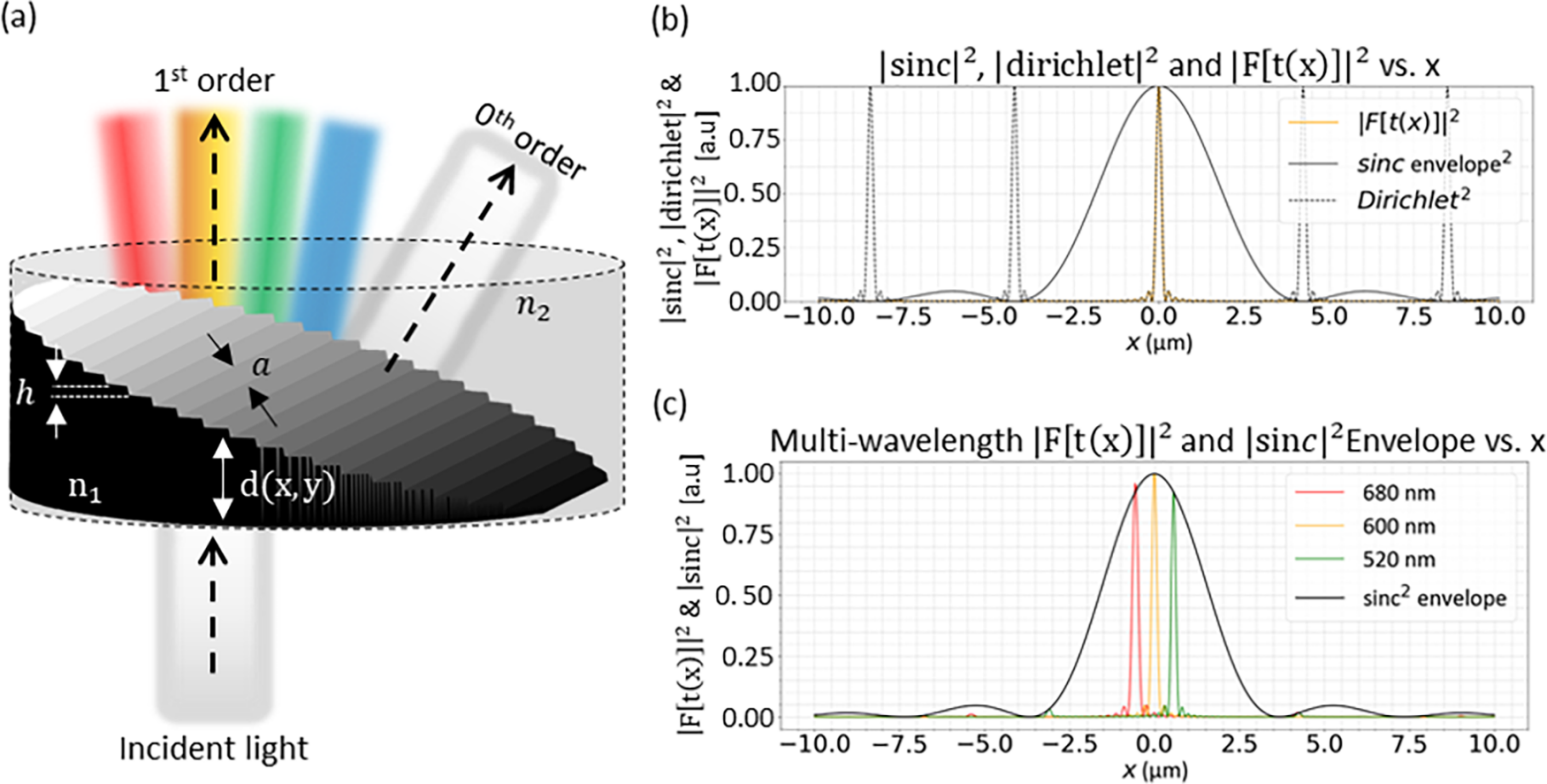
Terrace grating layout and simulations. (a) Schematic layout and parameters for normally incident visible light. *n*_1_ and *n*_2_ are the refractive indices of the two layers, h is the step height and a is the step width. At the blaze wavelength, the first order propagates on axis while the zeroth order is diffracted. (b) Simulations of optimal case for λ = 600 nm, Δ*n* = 0.006, *h* = 100 μm and *a* = 283 μm. The diffracted first order is aligned with the maximum of the sinc envelope, yielding 100% theoretical efficiency. (c) Simulated diffraction for three representative wavelengths near 600 nm. Although only the central wavelength is strictly optimal, neighboring wavelengths remain within the main lobe and maintain high efficiency.

For a given incident field at the BFP *U*_0_bfp__(*x*_bfp_,*y*_bfp_|*x*_*o*_,*y*_*o*_), the field after the mask is the product, and the corresponding field in image plane is

$$U_{i}\left(x_{i},y_{i}|x_{o},y_{o}\right)\propto F\left\{{U_{0}}_{bfp}\left(x_{bfp},y_{bfp}|x_{o},y_{o}\right)\cdot t\left(x_{bfp},y_{bfp}\right)\right\}\left(\frac{x}{\lambda f_{tube}},\frac{y}{\lambda f_{tube}}\right)=F\left\{{U_{0}}_{bfp}\left(x_{i},y_{i}|x_{o},y_{o}\right)\right\}\left(\frac{x}{\lambda f_{tube}},\frac{y}{\lambda f_{tube}}\right)⊗F\left\{t\left(x,y\right)\right\}\left(\frac{x}{\lambda f_{tube}},\frac{y}{\lambda f_{tube}}\right)$$ 5

where ⊗ denotes convolution, λ is the wavelength and *f*_tube_ is the focal length of the tube lens in the microscope, and the intensity in image plane, which, for imaging a point-source, is the PSF, is proportional to the absolute value squared

6 $$I\propto {\left|U_{i}\left(x_{i},y_{i}|x_{o},y_{o}\right)\right|}^{2}$$

The first term of the convolution is simply the system PSF without the phase mask, including aberrations, linear phase for off axis or spherical phase for defocused emitters. In the aberration-free case, this term reduces to the Airy disk. The second term of the convolution is the scaled Fourier transform of the mask complex amplitude transmittance function. The Fourier transform of eq 2 has an analytic solution (see SI Section 1)­

$$F\left[t\left(x,y\right)\right]\left(\frac{x}{\lambda f_{t\mathrm{ube}}},\frac{y}{\lambda f_{\mathrm{tube}}}\right)=a\cdot \delta \left(y\right)\cdot \sin c\left(\frac{a}{\lambda f_{\mathrm{tube}}}x\right)\cdot \frac{\sin\left(\frac{2\pi }{\lambda }\left(\Delta \mathrm{nh}-\frac{x}{f_{\mathrm{tube}}}a\right)\frac{N}{2}\right)}{\sin\left(\frac{2\pi }{\lambda }\left(\Delta \mathrm{nh}-\frac{x}{f_{\mathrm{tube}}}a\right)\frac{1}{2}\right)}$$ 7

The field function in eq 7 can be separated into three terms. (i) A Dirac delta function δ­(*y*), indicating no effect on the PSF along the *y*-axis; (ii) A sinc envelope unaffected by step height or refractive index difference, centered at the origin and determining the intensity ratio between diffraction orders; (iii) A periodic Dirichlet kernel, defining the position and width of diffraction orders in image space. According to the kernel, the positions in object space that correspond to peaks in image space, occur when both sine arguments are integer multiplications of π

8 $$\frac{2\pi }{\lambda }\left(\Delta \mathrm{nh}-\frac{x}{f_{\mathrm{tube}}/M}a\right)\frac{1}{2}=\pi m,m=0,\pm 1,\pm 2,\cdot \cdot \cdot$$

$$x_{m}\left(\lambda \right)=\left(\Delta n\cdot h-\lambda m\right)\frac{f_{\mathrm{tube}}/M}{a}$$ 9

Here, we set *x* → *x·M*, to account for system magnificaiton. Plugging eq 9 into eq 7 shows that, for the blaze wavelength in diffraction order *m*, when λ_blazed_ satisfies the condition Δ*n*·*h/m* = λ_blazed_, the lateral shift *x*_*m*_ nullifies and there is no deflection, unlike traditional blazed gratings. In addition, at this wavelength, the sinc envelope reaches its maximum, while other diffraction orders at this wavelength are not within the main lobe of the sinc function (Figure 1b). Thus, the order m diffraction has theoretical efficiency of 100%. Figure 1c shows simulated diffraction orders of three wavelengths around the blaze wavelength. Within this ∼160 nm range, the spots remain near the envelope center, retaining >90% efficiency. For clarity, the figure shows the envelope calculated at the central wavelength.

The step width *a* functions as the grating period and sets the spectral shift. The lateral shift Δ*x*_*m*_ in the image plane between two wavelengths, λ_1_ and λ_2_, at diffraction order *m* is

10 $$\Delta x_{m}=x_{m}\left(\lambda_{2}\right)-x_{m}\left(\lambda_{1}\right)=\left|\left(\lambda_{1}-\lambda_{2}\right)m\right|\frac{f_{\mathrm{tube}}}{a}$$

11 $$a=\left(\lambda_{1}-\lambda_{2}\right)\frac{f_{\mathrm{tube}}}{\Delta x_{m}}m$$

eq 11 therefore provides a direct, linear relation between Terrace step width and spectral displacement. Note that physical constraints, e.g., whether the component can be inserted into the available slot, are affected also by the total number of steps required to fill the diameter in the BFP (see SI Sections 2.1 and 2.2) and need to be considered in the design. However, due to the periodicity of the Terrace grating, mechanical registration is not required. As long as the element fully covers the objective’s BFP, the same mask can be used with different objectives while the dispersion will change as a function of magnification.

These relations between grating geometry, refractive index mismatch, wavelength, and diffraction efficiency guide the design of Terrace gratings. In the following subsections, we apply them to realize both a Single-order configuration, which exhibits a strong first order and high signal-to-noise ratio (SNR), and a Dual-order configuration, which exhibits a spectrally insensitive zeroth order that serves as a reference point in addition to the first spectral order.

### Single-Order Terrace Grating

Using eqs 9 and 11, one can select Terrace parameters suited for a given application. The step width *a* directly sets the spectral separation in image space, while the step height *h* and the refractive index mismatch Δ*n* define the blaze wavelength; this is the wavelength for which the design yields a spectral shift component with high efficiency in a single diffraction order, whereas energy of other orders attenuates almost completely. This enables the design of Single-order Terrace gratings, where, within a designed wavelength range, there is mostly a single, bright diffraction order in the image plane. In this work, we focus on the first diffraction order, which provides a more compact geometry.

### Dual-Order Terrace Grating

Although the Single-order Terrace design of the Terrace grating achieves high efficiency and, in theory, does not waste photons (aside from Fresnel and material losses) and slightly spreads the PSF over the FOV, it lacks a built-in reference point; this means that many imaging applications would suffer from ambiguity between positional and spectral information. Decoding spectral information in this case requires prior knowledge of the PSF displacement, which is only feasible in certain applications.

To address this limitation, by exploring eq 7, we designed the Dual-Order Terrace Grating, in which the refractive index mismatch is chosen such that both zeroth and first diffraction orders are visible. Since the zeroth order does not depend on the wavelength, it serves as a reference point and the lateral distance between the orders, *d*(λ), is proportional to the wavelength.

To find the geometry that results in a balanced PSF, from eq 7, we define the ratio between first and zeroth orders, which depends solely on the sinc envelope

$$\frac{I_{m=1}\left(\lambda \right)}{I_{m=0}\left(\lambda \right)}=\frac{\sin c^{2}\left(\frac{a}{\lambda f_{\mathrm{tube}}}x_{1}\right)}{\sin c^{2}\left(\frac{a}{\lambda f_{\mathrm{tube}}}x_{0}\right)}$$ 12

For the balanced case across the spectral range [λ_1_,λ_2_], the best values of *h*·Δ*n* are obtained when the inverse of the ratios are equal at both ends of the range

13 $$\frac{I_{m=1}\left(\lambda_{1}\right)}{I_{m=0}\left(\lambda_{1}\right)}=\frac{I_{m=0}\left(\lambda_{2}\right)}{I_{m=1}\left(\lambda_{2}\right)}$$

This condition ensures that the zeroth order at one spectral end is equal to the first order of the opposite end while intermediate wavelengths yield more balanced ratios. Moreover, the measured intensity ratios between the two orders can provide additional spectral information.

### Terrace Grating Integrated with 3D-Encoding Phase Masks

In Fourier optics scalar theory, when *N* phase masks are placed in sequence, the combined phase pattern can be written as the simple sum of their phase functions

14 $$t_{\mathrm{total}}\left(x,y\right)=\prod_{n=1}^{N}t_{n}\left(x,y\right)=\prod_{n=1}^{N}e^{i\phi_{n}\left(x,y\right)}=e^{i\sum_{n=1}^{N}\phi_{n}\left(x,y\right)}$$

15 $$\phi_{\mathrm{total}}\left(x,y\right)=\sum_{n=1}^{N}\phi_{n}\left(x,y\right)$$

Thus, the combined phase pattern ϕ_total_(*x*,*y*) can be realized by direct summation of the phase pattern, and the functionality of both elements is preserved.

Importantly, the simple, flat geometry of the Terrace grating makes it highly compatible with such integration, and continuous spectral encoding can be added to essentially any DOE that is suitable for fabrication.

The most straightforward configuration is simply to add a Dual-order Terrace to an existing design, e.g., a 3D encoding phase mask. In this case, each emitter produces two PSFs dictated by the original DOE, with their relative displacement encoding the spectral information. More sophisticated integrations, however, can yield even more compact and application-oriented designs, as we show in the “Spectrally Continuous Depth Encoding Phase Masks” section.

## Fabrication

All masks in this work were fabricated using 3D printing combined with near-index matched polymers.^9^ This approach allows scaling up the axial dimension of the element by orders of magnitude compared to standard approaches, e.g., photolithography, which enables fabrication using commercial 3D printers, while maintaining high optical quality. A simple flowchart of the process is shown in Figure 2.

**Figure 2:**
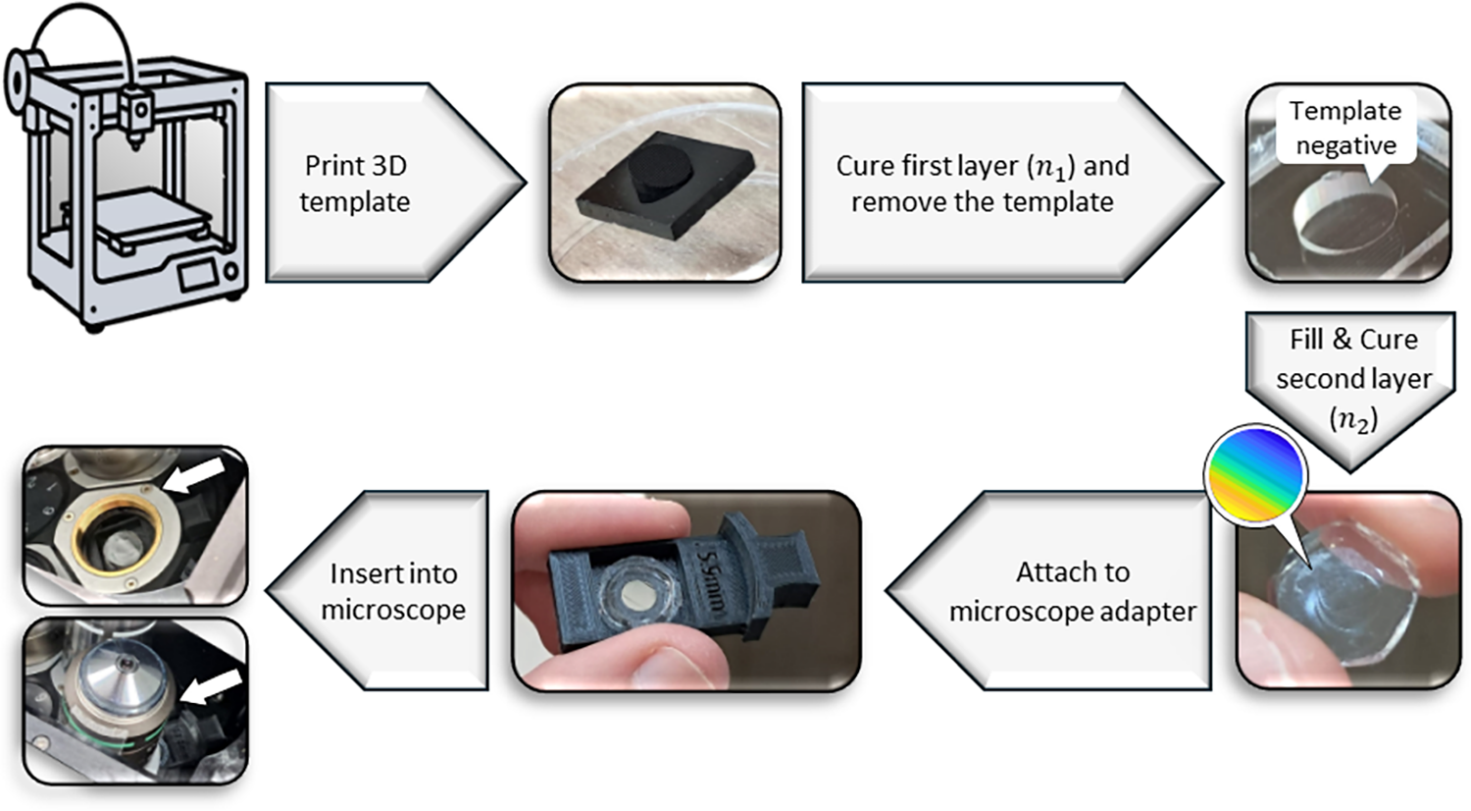
Fabrication process. The process begins with 3D printing of a template, followed by pouring and curing the first polymer layer, followed by removal of the template to obtain its negative shape. A second layer with a closely matched refractive index is then added to the negative template. The two layers are sandwiched between flat glass substrates. Finally, the completed mask is bonded to a 3D-printed adapter, allowing simple plug-and-play insertion into a conventional inverted microscope (DIC slot), Nikon Eclipse Ti2 in this example.

First, a phase mask template is fabricated by Fabrica’s Giga 25vx high-resolution 3D printer (see SI Section 2.3 for printed template topography). The first polymer (Polydimethylsiloxane) is then poured over the template, cured at 75 °C for several hours, and removed to result in its negative shape, forming the first layer. A second layer (RTV 269) with a closely matched refractive index (Δ*n* ≈ 0.006) is then added. Finally, to ensure that both sides of the DOE, interfaced with the air, are of optical quality, the element is sandwiched between two flat glass substrates. This process yields a flat phase element, consisting of two layers of closely matched refractive indices, sandwiched between glass substrates.

Finally, the phase mask is mounted and glued to a 3D-printed adapter designed to fit the dedicated slot of a conventional Nikon inverted microscope (Nikon Eclipse Ti2). Importantly, the adapter can be easily redesigned to any geometry, potentially allowing compatibility with a wide range of commercial microscopes. Currently, the slot used was originally dedicated for differential interference contrast (DIC) components. Note that the element is not necessarily precisely in the BFP; however, due to the periodicity of the Terrace grating, there is no centering requirement, and simply covering the entire BFP is sufficient.

## Results

### mRNA Barcode Imaging

We first demonstrate a Single-order Terrace Grating designed for imaging stretched mRNA molecules tagged with fluorescent NanoString barcodes.^13,14^ These are labeled RNA probes that allow multiplexed detection of RNA targets without amplification, which enables direct measurement of mRNA expression levels using a relatively simple process. The different mRNA targets are encoded in a unique sequence of six labeled patches on the probe, each labeled with distinct fluorescent dye (AF488, Cy3, AF594, AF647 with emission peaks at: 520 nm, 570 nm, 617 nm, 670 nm respectively), generating hundreds of unique molecular identifiers. Recent work has demonstrated accurate readout of these four-colored barcodes with a single spectral image using a spectral imaging module.^6,14^ There, spectral dispersion was achieved by two motorized rotating prisms.^6^ Here, we simplify the setup by using a single, cost-effective diffractive element, in a plug-and-play configuration, minimizing photon loss and requiring no optical expertise.

Thus, we aim to separate four wavelengths between 520 to 680 nm, with a central wavelength of ∼600 nm. The optical setup we used is described in SI Section 3. Using eq 11 and eq (S11), the parameters were determined as follows: a step height *h* of 100 μm, and a refractive index mismatch Δ*n* of 0.006. To support the required four-wavelength separation, we chose a step width *a* of 283 μm, corresponding to a lateral separation of 1.2 μm in object plane between the two extreme peaks (AF488 and AF647). At this step width, the number of steps used to cover more than the BFP area is 21, and accordingly, the thickness of the entire component is ∼3 mm, which easily fits into the 4 mm high slot of our microscope (see SI Section 2).

We validated the design with four-color Tetraspeck beads (T7280) with emission peaks at 430 nm, 515 nm, 580 and 680 nm. Figure 3a shows the results from a single bead: a vertical spectral shift with ∼1.3 μm separation between the green and red emissions of the bead confirms that the step height is close to the design. The measured refractive index mismatch of Δ*n* = 0.0061 (SI Section 4.1) aligns with the weak observed zeroth order. The measured spectral efficiency described in SI Section 4.1 shows 23% photon losses due to fabrication imperfection, material and Fresnel loss.

**Figure 3:**
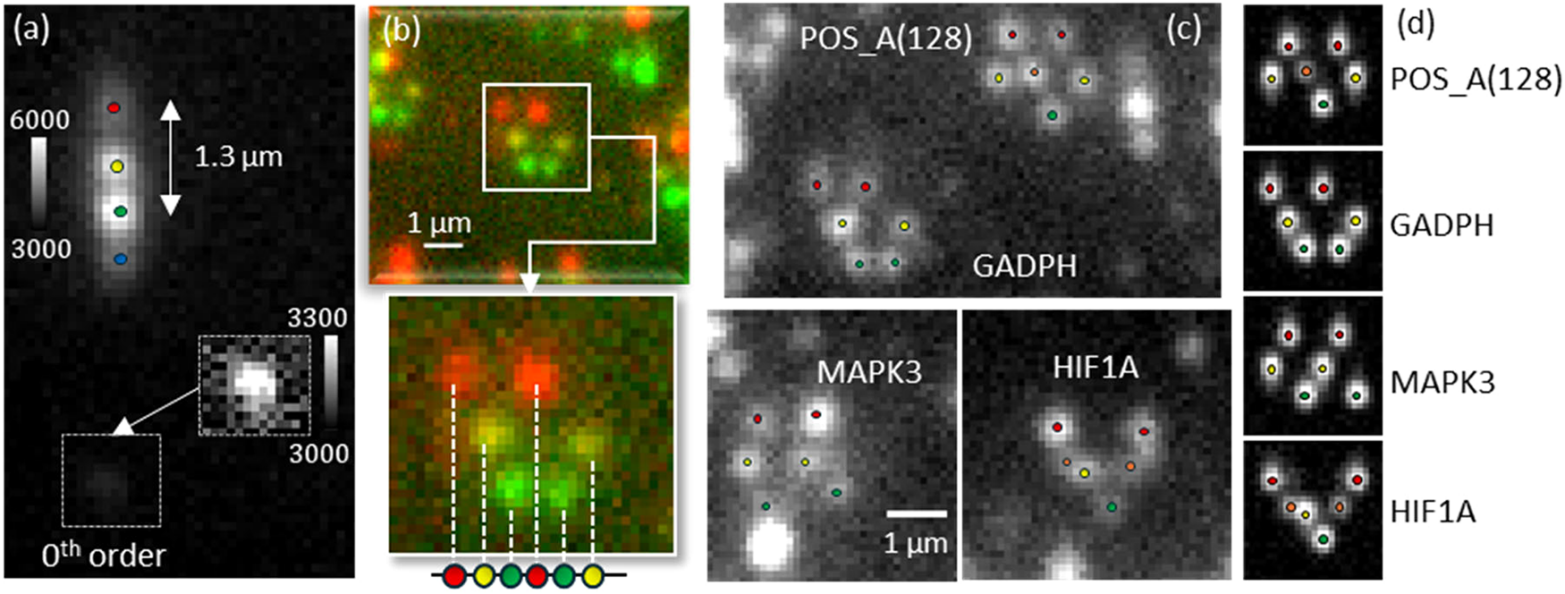
Experimental results of Single-order Terrace Grating. (a) Four-color Tetraspeck bead imaged with the Terrace grating zoom in and rescaling gray level on 0th order. The vertical spectral shift in the object plane matches the design, with ∼1.3 μm between green and red. The weak 0th order indicates that the step height and Δ*n* are as intended. Setup parameters are in SI Section 3. (b) False-color reference image of stretched mRNA molecules acquired by sequential laser illumination (top). The same molecules imaged with the Terrace grating wavelength-dependent vertical shifts consistent with reference (middle). Bottom- Sketch of the stretched molecule imaged without the mask. (c) Monochromatic snapshot of multiple barcoded molecules acquired with the Terrace grating. Four emitters are classified by vertical displacement and compared to simulation (d) of the four-color NanoStrings.

Next, we applied this plug-and-play Terrace component to snapshot imaging of stretched mRNA molecules (Figure 3b). To obtain a ground-truth measurement, we first used sequential illumination to generate false-color reference images. As expected, horizontally stretched mRNA molecules showed vertical shifts that correlated with the reference colors. The bottom panel of Figure 3b sketches the same molecule without the mask for comparison.

Finally, we simultaneously illuminated the sample to acquire a monochromatic snapshot with our spectral encoder and compared it to simulations, as well as to similar previously documented measurements.^14^ Figure 3c shows four classes of molecules imaged in one field of view, with emitters classified by vertical displacement. The results closely matched four-color barcodes reported in the simulations (Figure 3d). The transmission windows in the optical system are dominated by the dichroic mirror (Chroma TRF 89902-NK Dichroic mirror). Note that the yellow and orange dyes (Cy3 and AF594) share the same window (see SI Section 5), hence, the spectral separation is small. This can be easily improved using a suitable dichroic mirror or using a mask with larger separation.

Thus, by simply plugging in the 3D-printed Single-order Terrace Grating, we could identify distinct barcodes, with high resemblance to the expected barcodes, on a conventional microscope equipped only with a monochrome camera. This provides a simplified and accessible platform for snapshot, high-throughput barcode decoding that can be used even by nonexpert users.

### *C. elegans* Neuronal Imaging

We used the Dual-order Terrace to image NeuroPAL worms,^15^ in which all neurons within the*C. elegans*nervous system are uniquely and spatially identified by a specific set of fluorescent proteins. In this case, and in many others, simultaneous illumination and single image acquisition is extremely advantageous, as it enables imaging of dynamic scene and can increase throughput.

In our experiment, three excitation lasers (405, 488, and 561 nm) simultaneously excited mTagBFP2, CyOFP1 and mNeptune2.5, yielding estimated readout wavelengths near 460, 590, and 650 nm respectively, set by system transmission and fluorophore emission. Using eq 13, and the existing Terrace template (100 μm step height), we selected a refractive-index mismatch value that produced two diffraction orders with similar intensities across the visible spectrum (Δ*n* ∼ 0.0027, obtained by a combination of the materials^9^), allowing us to reuse the same printed template. The experimentally obtained refractive index was Δ*n* = 0.0034, which shifted the balance toward the first order at shorter wavelengths and reduced the blue zeroth-order intensity, while still maintaining sufficient SNR in both spots for our application (see SI Section 4.2 for further information).

For spectral calibration, we measured Tetraspeck beads (T7280) by sequential illumination with the three lasers (Figure 4a, left). Note that the zeroth order (wavelength-independent) serves as a reference, while the first order carries spectral information via the displacement *d*(λ). A vertical intensity profile (Figure 4a, center) presents *d*(λ). Based on the specification, we assume peak bead emissions at 515, 580, and 680 nm (Figure 4a, right) we fit a calibration curve λ_measured_(*d*) = 125.3·*d* + 55.9 [nm], where *d* is distance in object space in microns.

**Figure 4:**
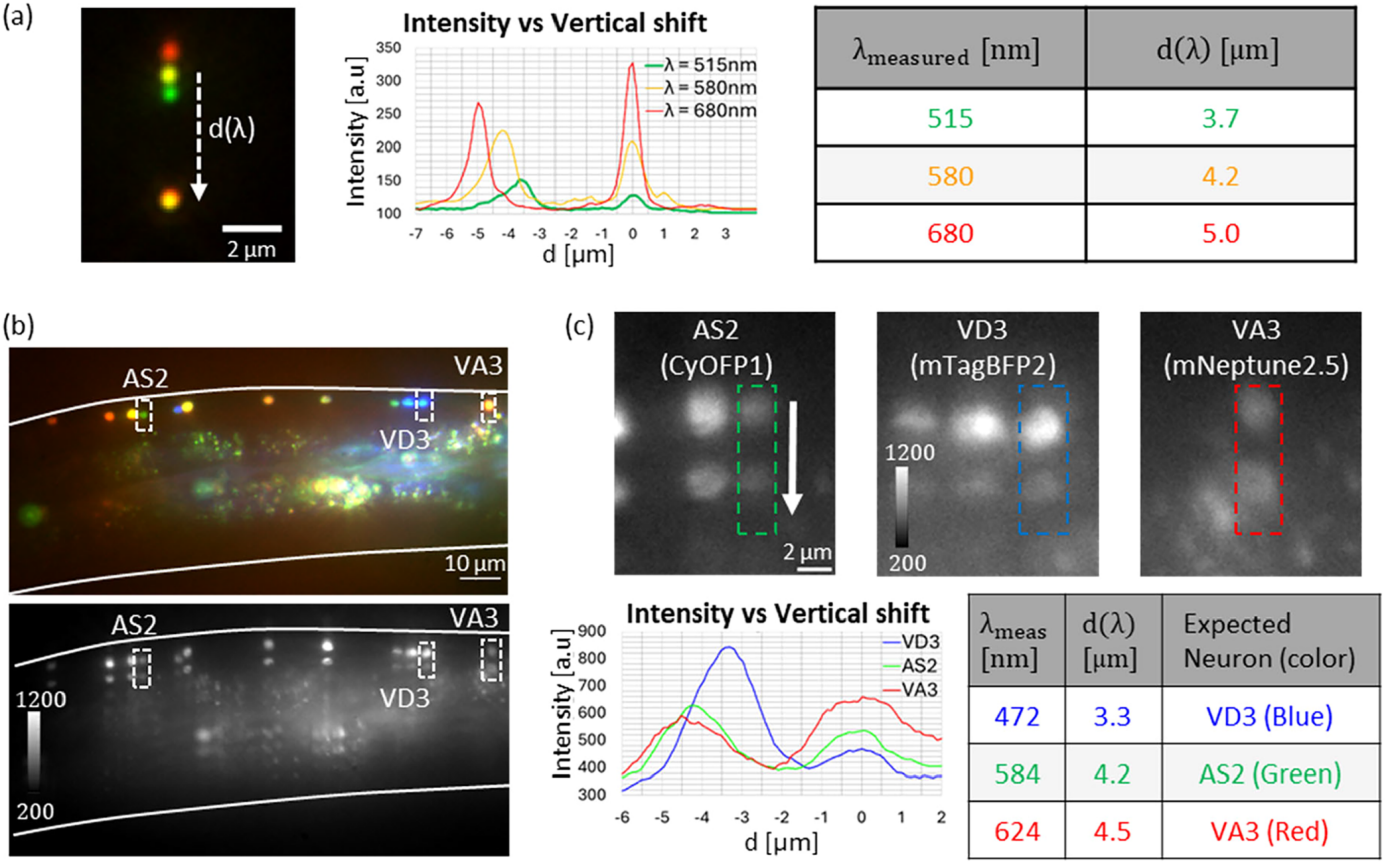
Snapshot imaging and spectral reconstruction of NeuroPAL worms using a Dual-order Terrace grating. (a) spectral calibration measurements using Tetraspeck beads (T7280). Left–false-color bead images at estimated emission wavelengths of 515, 580, and 680 nm (shown in green, yellow and red, respectively), acquired sequentially with 488, 561, and 640 nm excitation. The distance between 0th order replica (bottom) and 1st order replica (top), *d*(λ), depends on the wavelength. Center–vertical profile for the three emission wavelengths with origin at 0th order. Right–Calibration table of *d*(λ). (b) false-color image of the scene without the mask (top, sequential imaging) and with the Dual-order Terrace grating inserted (bottom, snapshot). Neurons around the ventral nerve cord were identified via their spectrum using our calibration, by comparison with the NeuroPAL atlas. (c) Top–zoom-in ROIs showing the VD3 (mTagBFP2, blue), AS2 (CyOFP1, green) and VA3 (mNeptune2.5, red) neurons. The upper replica is the first order, while the lower is the zeroth order. Bottom left–vertical intensity profiles the neurons common with origin at 0th order. Bottom right–table listing measured distance *d*(λ), the corresponding wavelength extracted from the calibration, λ_measured_, and the expected color based on NeuroPAL atlas.

Next, we acquired a reference image of the worm without the mask using sequential color acquisition, shown on the top panel in Figure 4b, and identified neurons around the ventral nerve cord by comparison to the NeuroPAL atlas.^15^ For demonstration, we focused on the VD3 (blue, mTagBFP2), AS2 (green, CyOFP1), and VA3 (red, mNeptune2.5) Ventral nerve cord neurons, each exhibiting a dominant emission band. After inserting the Dual-order Terrace and reacquiring the scene (Figure 4b, bottom), every neuron produced a duplicated signal: a wavelength-independent zeroth order and a spectrally shifted first order.

We then analyzed vertical profiles in selected regions of interest (ROIs) (Figure 4c), where the upper replica corresponds to the first order and the lower to the zeroth order. From the measured inter-replica distance *d*(λ) (Figure 4c, bottom-left) and the calibration curve, we extracted the corresponding wavelengths that classify VD3 as blue, AS2 as green and VA3 as red (Figure 4c, bottom-right).

The Dual-order Terrace grating enabled snapshot spectral measurement of NeuroPAL neurons, allowing instantaneous neuron identification that agrees with the NeuroPAL atlas. We note that the emission from the VA3 neuron, marked with red mNeptune2.5 fluorophore was partially cut by our system’s transmission window (Chroma TRF 89902-NK Dichroic mirror and Andor’s SONA-2BV11 EMCCD camera).

### Spectrally Continuous Depth Encoding Phase Masks

Depth encoding phase masks, such as Tetrapod^16,17^ or double-helix patterns,^18–20^ are used in 3D microscopy for high-precision volumetric localization, by encoding depth directly into the PSF shape. In this approach, a phase altering element, usually implemented by a DOE or a liquid crystal spatial light modulator, is placed at the system’s Fourier plane, changing the PSF shape as a function of the object’s distance from the focal plane. The resulting images are then analyzed with localization algorithms to recover the object’s lateral and axial coordinates. So far, when this approach was combined with spectral encoding, i.e., a single phase mask that encodes depth and color, it was designed for a discrete set of wavelengths (e.g., 2–3 fluorophores);^7,8^ thus, intermediate wavelengths produce inefficient and practically unusable PSFs. This discretely spectral approach enables simultaneous 3D localization and color separation, but at the cost of losing spectral information outside the targeted design bands.

Building on the section “Terrace Grating Integrated with 3D-Encoding Phase Masks”, we integrated our Terrace grating with two 3D-encoding phase masks, Tetrapod and double-helix (DH), achieving continuous spectral encoding, by two different methods. The composite masks were fabricated and inserted behind the objective as before. For the spectrally-encoded Tetrapod, as in eq 15, we simply summed the Dual-order Terrace and a Tetrapod phase map as shown in Figure 5a, resulting in a combined phase mask, a spectral Tetrapod mask (the “Spectralpod”). The three left columns of Figure 5b show single color z-stacks of Tetraspeck beads acquired with this mask: each point source splits into two Tetrapod PSFs; a wavelength-independent zeroth order (reference) and a spectrally shifted first order. The right column merges the three colors, highlighting that the inter-replica distance *d*(λ) encodes wavelength, while the Tetrapod shape encodes depth.

**Figure 5:**
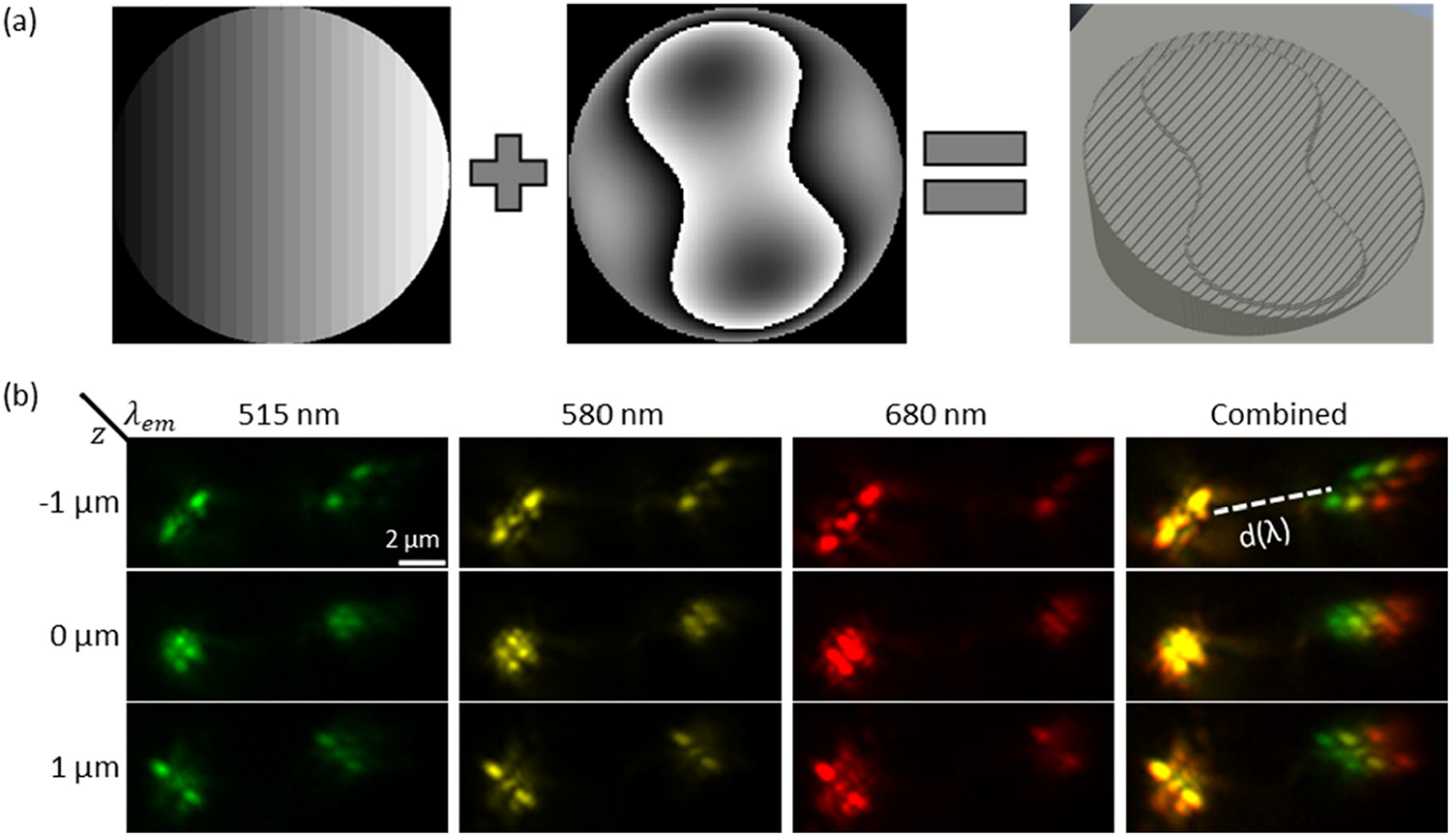
Z-stack of Spectralpod phase mask. (a) combination of Dual-order Terrace grating (left) with Tetrapod phase mask (center) yields a combined Spectralpod phase mask (right). (b) The measured multicolor PSF z-stack. Three false-color measurements of Tetraspeck beads with estimated emission wavelengths of 515, 580, and 680 nm (shown in green, yellow and red in the three left columns, respectively), were acquired sequentially using 488, 561, and 640 nm excitation. The right column shows the merged spectral measurements, where spectral information is encoded by *d*(λ): the distance between the 0th order Tetrapod PSF (left replica) and the 1st order PSF (right replica).

As with the Dual-order Terrace, the central wavelength and spectral separation are set by the geometry of the element. Because the Terrace provides continuous dispersion, *d*(λ) ∝ λ across the operating range, any wavelength within the band has an associated shift.

Next, we introduce the multispectral double-helix (Spectral-helix), which was inspired by the work of Ghanekar et al.,^22^ which exploits the double-helix’s property of lobe separability, by polarizing each lobe in the PSF with different polarization to measure them independently. In the Spectral-helix phase mask presented in Figure 6, we encode spectral information by integrating the Single-order Terrace Grating with opposite orientation on the two lobes, yielding mirrored color shift. Practically, the DH phase mask was split into two halves and Terrace steps with opposite directions were added, forming a wedge-like triangular-prism shape. The DH masks, prism-like Terrace steps, and the resulting Spectral-helix height map are shown in Figure 6a. The multicolor PSF z-stack in Figure 6b indicates that both spectral information and 3D information can be extracted by the combination of the distance and the angle between the lobes, with high photon efficiency thanks to the Single-order Terrace configuration and the relatively compact PSF. The z-projection in Figure 6c of the three wavelengths summarizes the wavelength-dependent PSF behavior across the axial range.

**Figure 6:**
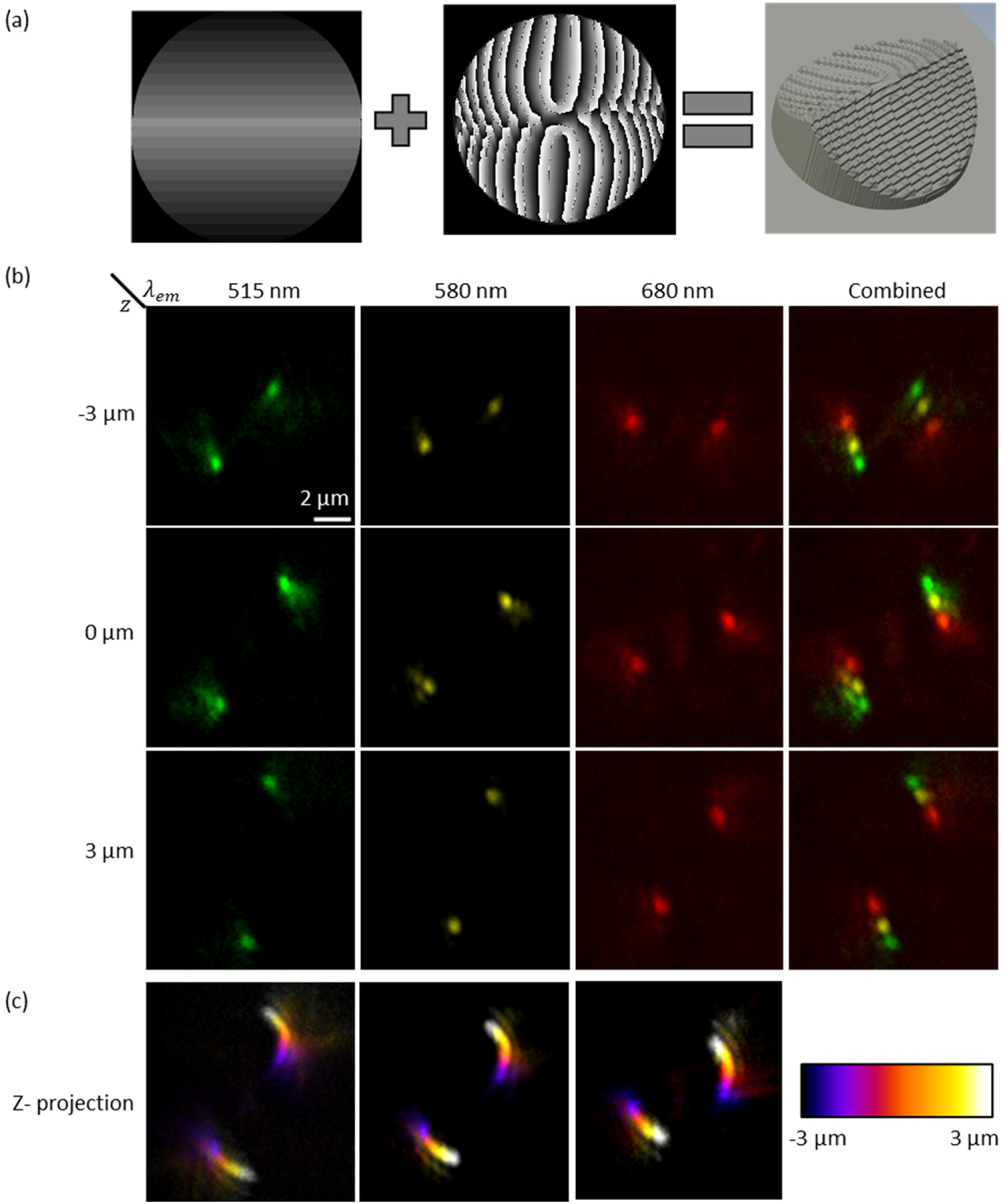
Z-stacks with a Spectral-helix phase mask. (a) Combination of the two mirrored Single-order Terrace Grating (left) with a DH phase mask (center) yields a Spectral-helix mask (right). (b) Measured multicolor PSF z-stack: three measurements of Tetraspeck beads with estimated emission wavelengths of 515, 580, and 680 nm (shown in green, yellow and red in the three left columns, respectively), acquired sequentially with 488, 561, and 640 nm excitation. The right column shows the merged spectral measurement. Spectrum and depth are encoded together by the interlobe distance and the lobe rotation. (c) Z-projection: for each emission wavelength, a sum projection of the PSF across the axial range is shown. Color encodes axial position (depth) according to the color-bar at right, presenting the Spectral-helix depth profile and the wavelength-dependent behavior.

Although the spectral Z-projections show clear differences over an axial range of 6 μm, there is a short axial interval where separating wavelengths is ambiguous (see SI Section 6 for more information). This can be mitigated using prior information, e.g., tracking the same emitter over time, or using an elaborate PSF model that considers the precise dependence of the PSF shape on spectrum.^7,8^ As with the Spectralpod and Terrace grating, despite these short axial regions of ambiguity, every wavelength within the operating band maps to a unique PSF.

## Conclusions and Discussion

We have developed and demonstrated a compact and versatile diffractive element for spectral encoding in microscopy, namely the Terrace grating. Its flat, simple geometry is compatible with plug-and-play 3D-printing-based fabrication, enables straightforward integration with other phase masks, and, in the common case of widefield illumination, maintains the desired illumination path and angle. The spectrally encoded PSF that is obtained is relatively compact, enabling imaging of dense samples at high SNR, expanding the work on compact multispectral imaging.^23–26^

We realized three configurations: (i) Single-order Terrace Grating, concentrating photons into a single diffraction order; (ii) Dual-order Terrace grating, which introduces a wavelength-independent reference order; and (iii) the spectral 3D phase masks family, which integrates Terrace encoding into engineered PSFs for volumetric imaging. Together, these implementations highlight how simple, flat geometry can provide multiple and continuous encoding strategies tailored to different applications.

We used the Single-order Terrace Grating for snapshot decoding of stretched mRNA barcodes, with results closely matching the NanoString fluorescent barcodes analyzed in previous work,^14^ albeit using a simpler setup, necessitating only a single cost-effective element plugged into an existing slot in the microscope.

The Dual-order grating was used for snapshot spectral imaging of NeuroPAL worms, where the zeroth order served as a stable reference and the displaced first order encoded spectral identity. We reconstructed the expected color codes and identified neurons around the ventral nerve cord. This method can be better improved by achieving more uniform illumination, better algorithms and more data.

Finally, we integrated the Terrace grating with depth-encoding phase masks. The Spectralpod (Dual-order Terrace + Tetrapod) preserves Tetrapod depth encoding while adding a continuous spectral shift: each emitter yields a wavelength-independent zeroth order and a spectrally shifted first order Tetrapod. The Spectral-helix uses a Single-order Terrace with opposite orientations on the two DH lobes to produce mirrored color shifts. Wavelength and depth are decoded from the interlobe distance and the lobe rotation. We illustrated the wavelength-dependent behavior and uniqueness across most of the axial range. Both spectral 3D phase masks have surprisingly simple geometry.

Despite these advances, several limitations remain. While we demonstrate robust performance at representative wavelengths, broader characterization is needed to quantify efficiency and field of view-dependence (SI Section 7). Our 3D-printing + near-index-matching pipeline enables rapid, low-cost prototyping, but improvements in surface quality, material stability, and AR coatings would further reduce photon losses. Finally, the total height required for large spectral separations at small Δ*n* can be a drawback.

In conclusion, Terrace grating optics offers a practical, flexible alternative to conventional blazed gratings and prism-based dispersion. It combines ease of fabrication, plug-and-play integration with standard microscopes, and compatibility with depth-encoding phase masks to deliver continuous spectral encoding and can provide simultaneous color and depth readout: a path toward simplified yet powerful spectral imaging in biological microscopy.

## Methods

### NanoString Barcode Sample Preparation

We used the same NanoString cartridge that was described previously.^14^ Briefly, native RNA samples were prepared according to the manufacturer’s instructions using the nCounter Human Inflammation V2 Panel (NanoString) codeset. To remove all fiducial markers for spectral imaging, we extracted all the imaging buffer containing the fiducial markers from one of the reagent plates prior to loading it onto the prep station. Samples were then loaded onto the prep station and incubated under high sensitivity program for 3 h. Following the prep station, the cartridge was loaded with imaging buffer that did not contain fiducial-marker beads.

### PSF Simulations

NanoString barcode simulations were performed by a custom Matlab code. Here we provide a short description of the pipeline:

1. Excitation and emission spectra of four commercial fluorophores together with our dichroic mirror (Chroma TRF 89902-NK). The 4-color barcodes simulations were downloaded from Semrock’s SearchLight spectra viewer. Each of the fluorophores’ spectrum was multiplied by the filter’s spectrum to produce the actual spectrum imaged on our camera.
2. The wavelength to pixel displacement calibration curve was calculated according to eq 10.
3. Barcode fluorophore combinations were then simulated by converting each fluorophore’s spectrum into a diffraction limited image. This was done by assigning a Gaussian with unity amplitude and 1.2-pixel standard deviation to each wavelength in the emission spectrum. Each Gaussian was displaced according to the eq 9 and summed together with other gaussians. Finally, the total summed intensity of all gaussians was normalized to unity and multiplied by an excitation efficiency factor which was calculated by the excitation spectrum value (fractions only) at the excitation laser wavelength.
4. This process was repeated for the selected fluorophores sequences at the six barcode positions, and all images were summed to provide the barcode’s spectral image.
5. To further resemble the experimental images, a noise model was added to all simulated spectral PSF images using the imnoise function in Matlab. The noise model used in this work was a sum of a poisson distributed shot-noise and Gaussian noise with a constant mean of 0.3 and 0.000625 variance.

### NeuroPAL Worm Sample Preparation

For neuronal imaging, individual NeuroPAL worms^15^ were mounted on a slide with a 2% agar pad and immobilized using 5 mM tetramisole in M9 buffer.

## Supplementary Material





## Data Availability

CAD drawing of Terrace grating are available free of charge. All other CAD drawings available upon request.

## Notes

Terrace grating 100 um diameter 6 mm (ZIP)
Additional information on the imaging model, mask physical dimensions and real dimensions of the fabricated template, the optical setup, efficiency measurement of fabricated Single-order mask, Dual-order ratio measurement of fabricated mask, dichroic mirror transmission window, spectral double-helix ambiguity and field-of-view dependence (PDF)
