# Supplementary material for: Compact Spectral Encoding Microscopy by Terrace Grating Optics
Source: ACS Photonics. 2026 Feb 16;13(5):1407–16. doi: 10.1021/acsphotonics.5c02701 (PMC12964586; doi:10.1021/acsphotonics.5c02701)
Supplement: Supplementary file 2 [file ph5c02701_si_002.pdf]

Supporting Information

Compact Spectral Encoding Microscopy by Terrace Grating Optics

ORI REFAEL COHEN[1], REUT ORANGE KEDEM[1], LEONID LEITES[1], AMIT PARIZAT[2], JONATHAN JEFFET[3], LIOR LAUFER[4], SHAY STERN[4], YUVAL EBENSTEIN[3] AND YOAV SHECHTMAN[1,2,5,*]

[1]*Russel Berrie Nanotechnology Institute, Technion–Israel Institute of Technology, Haifa 3200003, Israel*
[2]*Faculty of Biomedical Engineering, Technion–Israel Institute of Technology, Haifa 3200003, Israel*
[3]*Raymond and Beverly Sackler Faculty of Exact Sciences, Tel Aviv University, Tel Aviv 6997801, Israel*
[4]*Faculty of Biology, Technion–Israel Institute of Technology, Haifa 3200003, Israel*
[5]*Faculty of Electrical and Computer Engineering, Technion–Israel Institute of Technology Haifa 3200003, Israel*
**yoavsh@bm.technion.ac.il*

Supplementary Pages S1-S14

Supplementary Figures S1-S8

Supplementary Tables S1-S3

Table of Contents

Table of Contents 2
1. Imaging model 3
2. Mask dimensions 4
    2.1. Clear aperture (mask diameter) 4
    2.2. Total physical height 4
    2.3. Fabricated Template 5
3. Optical Setup 7
4. Efficiency and Dual-order ratio 8
    4.1. Single-order Mask Efficiency 8
    4.2. Dual-order ratio 9
5. Dichroic mirror Transmission window 10
6. Spectral Double-helix Ambiguity 11
7. Field-of-View dependence 12

1. Imaging model

In the "Imaging model" section in the main text, we defined the Terrace grating's complex amplitude transmittance function and use its Fourier-transform in the final imaging expression. In this section, we provide the full derivation of the closed-form solution of the Fourier transform used to obtain the imaging model, from the complex amplitude, step by step to the image-plane field. All symbols (e.g., $U_i, x_i, y_i, x_o, y_o, a, h, \Delta n, f_{tube}$) follow the main text.

Starting from the complex amplitude transmittance, written as a sum of shifted rectangles (see main text), the Fourier transform that needs to be calculated to derive the Terrace grating PSF is:

(S1) $$\mathcal{F}\{t(x,y)\}(v_x,v_y) = \mathcal{F}\left\{\sum_{m=-\frac{N-1}{2}}^{\frac{N-1}{2}} e^{i\frac{2\pi\Delta n}{\lambda}\cdot mh} \cdot rect\left(\frac{x-ma}{a}\right)\right\}(v_x,v_y)$$

The spatial frequencies, (v_x, v_y), are defined in terms of the physical dimensions of the system:

(S2) $$v_x = \frac{x_i}{\lambda f_{tube}}, \quad v_y = \frac{y_i}{\lambda f_{tube}}$$

Using linearity and the shift property of the Fourier transform in Eq. (S1):

(S3) $$\mathcal{F}\left\{\sum_{m=-\frac{N-1}{2}}^{\frac{N-1}{2}} e^{i\frac{2\pi\Delta n}{\lambda}\cdot mh} \cdot rect\left(\frac{x-ma}{a}\right)\right\}(v_x,v_y) = \sum_{m=-\frac{N-1}{2}}^{\frac{N-1}{2}} e^{i\frac{2\pi\Delta n}{\lambda}\cdot mh} \cdot \mathcal{F}\left\{rect\left(\frac{x-ma}{a}\right)\right\}(v_x,v_y)$$

The Fourier transform of the rectangular function is a sinc function:

(S4) $$\mathcal{F}\left\{rect\left(\frac{x-ma}{a}\right)\right\}(v_x,v_y) = e^{-i2\pi v_x ma} a \cdot sinc(a(v_x)) \cdot \delta(v_y)$$

Plugging Eq. (S4) into Eq. (S3), keeping only the terms that include the sum index m:

(S5) $$\mathcal{F}\{t(x,y)\}(v_x,v_y) = a \cdot \delta(v_y) \cdot sinc(a(v_x)) \cdot \sum_{m=-\frac{N-1}{2}}^{\frac{N-1}{2}} e^{i2\pi\cdot m\left(\frac{h\cdot\Delta n}{\lambda} - v_x a\right)}$$

The summation in Eq. (S5) is the Dirichlet kernel:

(S6) $$\sum_{m=-\frac{N-1}{2}}^{\frac{N-1}{2}} e^{i2\pi\cdot m\left(\frac{h\cdot\Delta n}{\lambda} - v_x a\right)} = \frac{sin\left(2\pi\left(h\cdot\frac{\Delta n}{\lambda} - v_x a\right)\frac{N}{2}\right)}{sin\left(2\pi\left(h\cdot\frac{\Delta n}{\lambda} - v_x a\right)\frac{1}{2}\right)}$$

Using Eq. (S6) in Eq. (S5), we obtain the closed-form of the transform:

(S7) $$\mathcal{F}\{t(x,y)\}(v_x,v_y) = a \cdot \delta(v_y) \cdot sinc(a(v_x)) \cdot \frac{sin\left(2\pi\left(\frac{h\cdot\Delta n}{\lambda} - v_x a\right)\frac{N}{2}\right)}{sin\left(2\pi\left(\frac{h\cdot\Delta n}{\lambda} - v_x a\right)\frac{1}{2}\right)}$$

To get the physical dimensions, we substitute the spatial frequencies in Eq. (S7), with the relations in Eq. (S2)

(S8) $$\mathcal{F}\{t(x,y)\}(x_i,y_i) = a \cdot \delta\left(\frac{y_i}{\lambda f_{tube}}\right) \cdot sinc\left(a\left(\frac{x_i}{\lambda f_{tube}}\right)\right) \cdot \frac{sin\left(\frac{2\pi}{\lambda}\left(h\cdot\Delta n - \frac{x_i}{f_{tube}}a\right)\frac{N}{2}\right)}{sin\left(\frac{2\pi}{\lambda}\left(h\cdot\Delta n - \frac{x_i}{f_{tube}}a\right)\frac{1}{2}\right)}$$

Finally, the electric field in image plane produced by a point-source at (x_o, y_o), is the native system's response convolved with the PSF caused by the Terrace phase modulation:

(S9) $$U_i(x_i, y_i | x_o, y_o) \propto$$

$$= \mathcal{F}\left\{U_{0_{bfp}}(x_i, y_i|x_o, y_o)\right\}\left(\frac{x}{\lambda f_{tube}}, \frac{y}{\lambda f_{tube}}\right) \otimes \left(a \cdot \delta\left(\frac{y_i}{\lambda f_{tube}}\right) \cdot sinc\left(a\left(\frac{x_i}{\lambda f_{tube}}\right)\right) \cdot \frac{sin\left(\frac{2\pi}{\lambda}\left(h \cdot \Delta n - \frac{x_i}{f_{tube}} a\right)\frac{N}{2}\right)}{sin\left(\frac{2\pi}{\lambda}\left(h \cdot \Delta n - \frac{x_i}{f_{tube}} a\right)\frac{1}{2}\right)}\right)$$

2. Mask dimensions

This section states the theoretical mechanical bounds on the lateral size and the total height of a Terrace grating mask. The Terrace parameters are tied to the PSF behavior derived in "Theory" section in the main text and to the required spectral displacement. We use the same notation as in the main text.

2.1. Clear aperture (mask diameter)

To maximize efficiency and minimize photon loss, the phase mask, which is placed near the objective's back focal plane (BFP), must cover the entire beam. Using the Abbe sine condition, the required clear diameter is

(S10) $$D_{mask} = 2f_{tube} \cdot \frac{NA}{\sqrt{M^2 - NA^2}}$$

where f_{tube} is the focal length of the tube-lens, M is the system magnification and NA is the objective's numerical aperture[1]. This diameter sets the minimum lateral extent that guarantees full pupil coverage.

2.2. Total physical height

For a Terrace template with step width (period) a and step height h, the total number of steps across the clear aperture is given by:

(S11) $$N = ceil\left\{\frac{D_{mask}}{a}\right\} = ceil\left\{2f_{tube} \cdot \frac{1}{a}\frac{NA}{\sqrt{M^2 - NA^2}}\right\}$$

where the "ceil" operator rounds the number up to the next integer. The corresponding total physical height (stack height) is:

(S12) $$h_{total} = h \cdot ceil\left\{2f_{tube} \cdot \frac{1}{a}\frac{NA}{\sqrt{M^2 - NA^2}}\right\}$$

This term is linked to the required spectral shift. Expressing the design in terms of the desired spectral displacement in the object plane, Δx_m, for diffraction order $m = 1$, using Eq (11) from the main text, we obtain:

(S13) $$h_{total} = h \cdot ceil\left\{2f_{tube} \cdot \frac{1}{(\lambda_1 - \lambda_2)\frac{f_{tube}/M}{\Delta x_1}}\frac{NA}{\sqrt{M^2 - NA^2}}\right\} = h \cdot ceil\left\{2 \cdot \frac{\Delta x_1}{(\lambda_1 - \lambda_2)}\frac{M \cdot NA}{\sqrt{M^2 - NA^2}}\right\}$$

At high magnification, $M^2 \gg NA^2$, Eq (S13) is simplified to:

(S14) $$h_{total} \approx h \cdot ceil\left\{2 \cdot \frac{\Delta x_1}{(\lambda_1 - \lambda_2)} NA\right\}$$

For the Single-order and Dual-order Terrace grating, this can also be directly related to the blaze wavelength in order $m = 1$, using the relation:

(S15) $$h = \frac{\lambda_{blazed}}{\Delta n}$$

Hence, the total height achieved by substituting Eq. (S15) into Eq. (S14) is:

(S16) $$h_{total} \approx \frac{\lambda_{blazed}}{\Delta n} \cdot ceil\left\{2 \cdot \frac{\Delta x_1}{(\lambda_1 - \lambda_2)} NA\right\}$$

Figure S1 illustrates a simulation of total phase mask height for $f_{tube} = 200$ mm, M =100, NA = 1.35 and h = 100 µm, for different spectral shifts Δx_1, over wavelength range $[\lambda_1, \lambda_2]$ at the object plane (λ_1=515 nm and λ_2= 680 nm). These parameters correspond to the Single-order Terrace grating, with blaze wavelength 600 nm and refractive index mismatch $\Delta n = 0.006$.

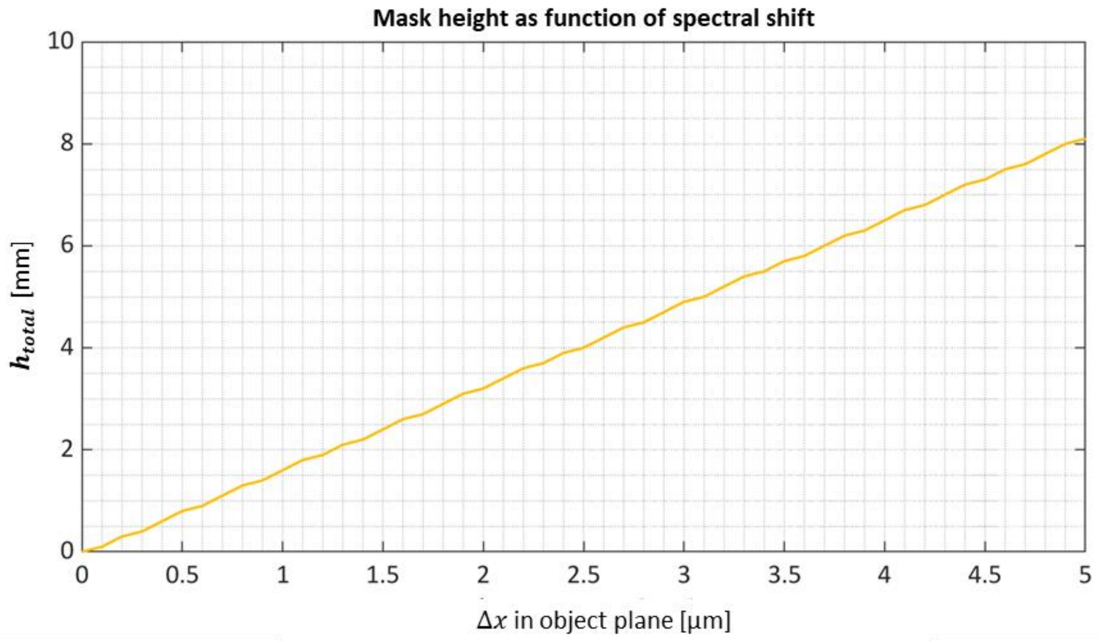

Figure S1. Maximum mask height $h_{\max}$ vs. desired spectral displacement Δx in object plane corresponds to our optical system (simulation). Parameters: $f_{\text{tube}} = 200$ mm, $M = 100$, NA = 1.35, step height $h = 100$ µm, blaze wavelength at $\lambda_{\text{blazed}} = 600$ nm and index mismatch $\Delta n = 0.006$ (Single-order design). For $\Delta x \approx 1.2$ µm over wavelength range [520,680] ($\lambda_1 = 520$ nm, $\lambda_2 = 680$ nm) the required height approaches ~2 mm. The height scales linearly with Δx and inversely with Δn.

Mask height in the case of spectral shift $\Delta x \approx 1.2$ µm, reaches 2 mm. This limitation is the main limit of spectral shift in the case of near-index-matched based Terrace gratings, where the height is scaled up significantly; in the case of Δn~0.5, on glass against air, the height would be smaller by two orders of magnitude but is much more challenging to fabricate due to optical roughness requirements (see comparison in section 2.3).

2.3. Fabricated Template

The templates have been printed on a Fabrica Giga 25vx printer (XY resolution up to 7.6 µm, accuracy ±5 µm and surface roughness ~0.9 µm). To validate the dimensions of the template used to fabricate the Single-order and Dual-order terrace grating, we measured it in a Sensofar S Neox 3D Optical Profiler. Figure S2 shows the height map and step indexing, while Table S1 lists, for each step, the mean height (center column) and the inter-step height ("step size", right column). The mean step width is 281.8 µm (16 steps across 4.5082 mm) and the mean step height is 95.4 µm. Roughness, R_a, estimated as the standard deviation of the steps height, measured with larger magnification over a smaller area, yields roughness < 0.4 µm.

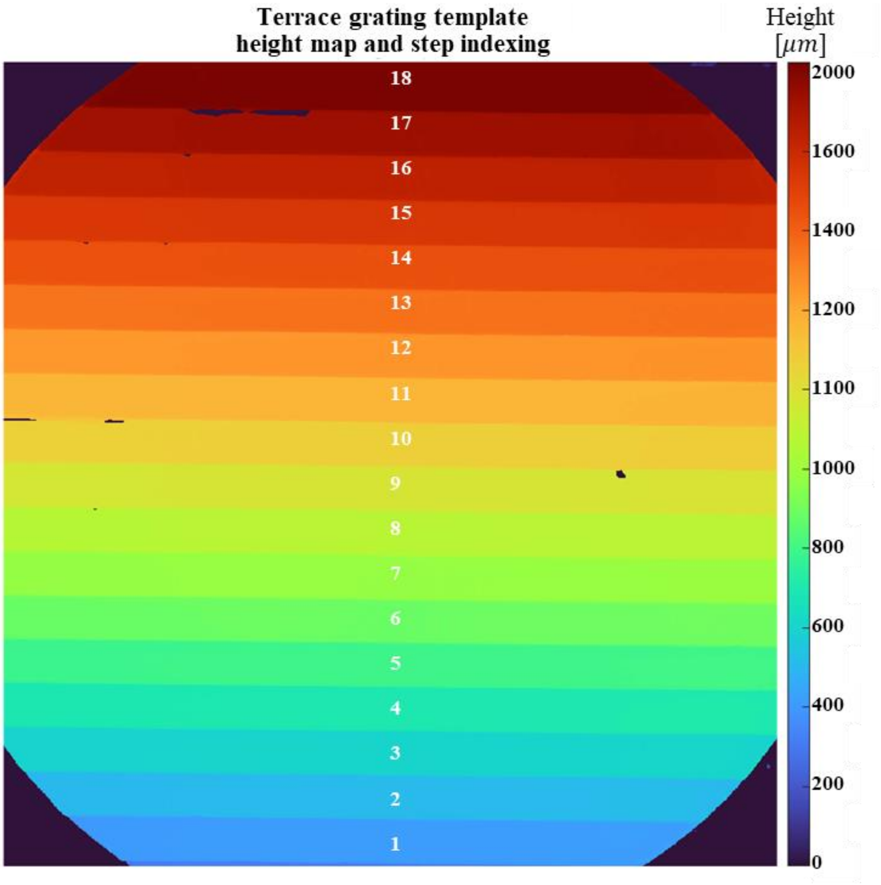

Figure S2. Height map and step indexing of terrace grating template, measured with Sensofar S Neox 3D Optical Profiler. Height values are in µm. Horizontal and vertical fields of view are 4.9209 mm and 5.1197 mm, respectively. Numerical values are given in Table S1.

step index	mean height [µm]	step size [µm]
1	494.9	-
2	590.2	95.3
3	685.2	95.0
4	780.6	95.4
5	876.5	95.9
6	970.7	94.2
7	1066.5	95.8

8	1163.1	96.6
9	1259.1	96.0
10	1353.9	94.8
11	1449.3	95.4
12	1544.6	95.3
13	1639.6	95.0
14	1735.0	95.4
15	1830.7	95.7
16	1926.5	95.8
17	2021.6	95.1
18	2116.3	94.7

Table S1. Measured steps. Left: step index (corresponds to labels in Figure S2). Center: mean height of each segment. Right: step size (difference between successive mean heights. The average step height is 95.4 µm.

According to Eq. (10) in the main text, step width deviations affect the spectral displacement in the image plane; here we see that these deviations introduce only a small deviation from design value (281.8 µm instead of 283 µm). The 5% deviation in step height (95.4 µm instead of 100 µm), multiplied by refractive index mismatch (Δn) sets the blaze wavelength and can be compensated by increasing Δn by ~5%. The measured roughness, with $\Delta n = 0.006$ corresponds to phase error $\phi_{\mathrm{err}} \approx R_a \Delta n \cdot \left(\frac{2\pi}{\lambda}\right) = 0.025\ \mathrm{rad}$, i.e. less than 1% of a 2π period, preserving good optical quality.

3. Optical Setup

The optical setup is presented in Figure S3. The microscope system is a commercial Nikon Eclipse Ti2 microscope with 200 mm tube lens and SR HP Plan Apo 100x/1.35 silicon immersion objective lens. Excitation was provided by a four-color laser at 405, 488, 561 and 640 nm and a multiband dichroic mirror (Chroma TRF 89902-NK) was used to filter out the excitation and transmit emission light. Images were acquired on an Andor SONA-2BV11 EM-CCD camera with 11 µm pixel pitch.

All Terrace-based phase masks were mounted in the infinity space between the objective and the tube lens through a DIC dedicated slot. During acquisition the sample has been placed at the objective working distance.

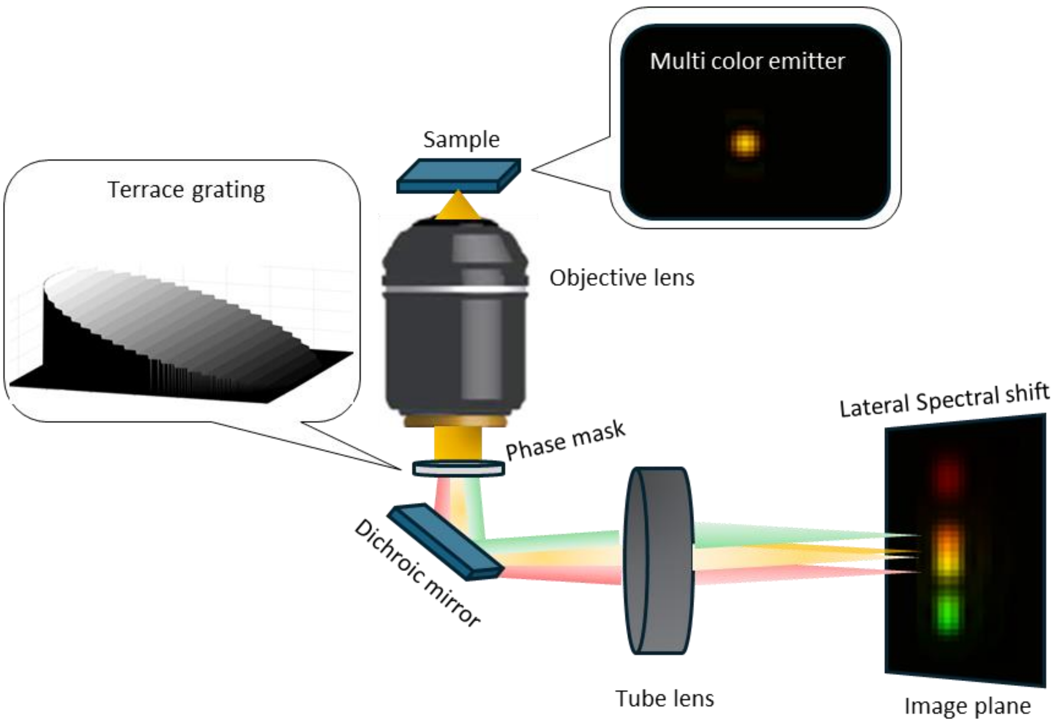

Figure S3 – Schematic of the optical setup. A four-color laser (405\488\561\640 nm) illuminates the sample through a multiband dichroic. Fluorescent emission is imaged by the 100×/1.35 objective and the 200 mm tube lens to the Andor SONA-2BV11 camera. Terrace-based phase masks are mounted in the infinity space (DIC slot) immediately behind the objective. When masks are in, a wavelength-dependent lateral spectral displacement is produced at the image plane, in addition to the depth encoding with the Spectralpod/Spectral-helix masks as described in the main text.

With the Terrace mask in place, a lateral spectral shift is produced at the image plane.

4. Efficiency and Dual-order ratio

In this section we quantify two performance parameters of the Terrace designs: (i) Single-order efficiency: the fraction of total intensity in the 1st order diffraction by the Single-order Terrace compared to the no-mask case; and (ii) the Dual-order ratio: the intensity ratio between 0th and 1st order on the Dual-order Terrace across wavelengths. This measurement can also be used for empirical refractive index mismatch estimation.

The measurement setup is composed of a collimated plane wave illuminating the mask, where beam diameter is set close to the mask diameter by an iris. A 200 mm singlet formed the Fourier plane on a camera, placed at its focal distance, so the recorded image is the mask's PSF. Excitation was applied sequentially with the three lasers 488/561/640 nm. For both simulations and measurements, total intensities were computed as integrals (summation) over the full lobe area**.**

4.1. Single-order Mask Efficiency

We compared the total intensity (gray levels) in the main lobe, measured with the Single-order Terrace, to the measured intensity without it. Results are listed in Table S2.

Measured values reflect the fabricated blazed-grating configuration as well as non-idealities: fabrication tolerances, Fresnel transmission/reflection losses, material absorption and scattering, etc, and the highest measured efficiency occurs at 640 nm. For refractive index estimation, we applied a single scale factor on the simulation, so that the 640 nm simulated lobe in Table S2 matches the measured value. Next, by comparing the efficiencies of each measured wavelength to the simulation, we could extract the refractive index that best matches

these values. Accordingly, The inter-channel ratios are best matched by a simulation with $\Delta n = 0.0061$ (target is 0.006). The resulting efficiencies at 640, 561 and 488 nm are 67.1%, 57.9% and 42%, respectively, as listed in Table S2.

λ	640 [nm]	561 [nm]	488 [nm]
Measured Efficiency [%]	67.1%	59.6%	41.3%
Simulated Efficiency [%] for $\Delta n = 0.0061$	67.1%	57.9%	42%

Table S2. Single-order Terrace grating efficiency. Ratio of integrated lobe intensity with mask / without mask for each wavelength. Simulated values are shown for the best-fit $\Delta n = 0.0061$ and are normalized so the simulated 640 nm matches the measurement.

The theoretical diffraction efficiency near the blaze wavelength is ~100% (Figure S4), so the observed ~33% loss at 640 nm is attributed to non-diffraction factors.

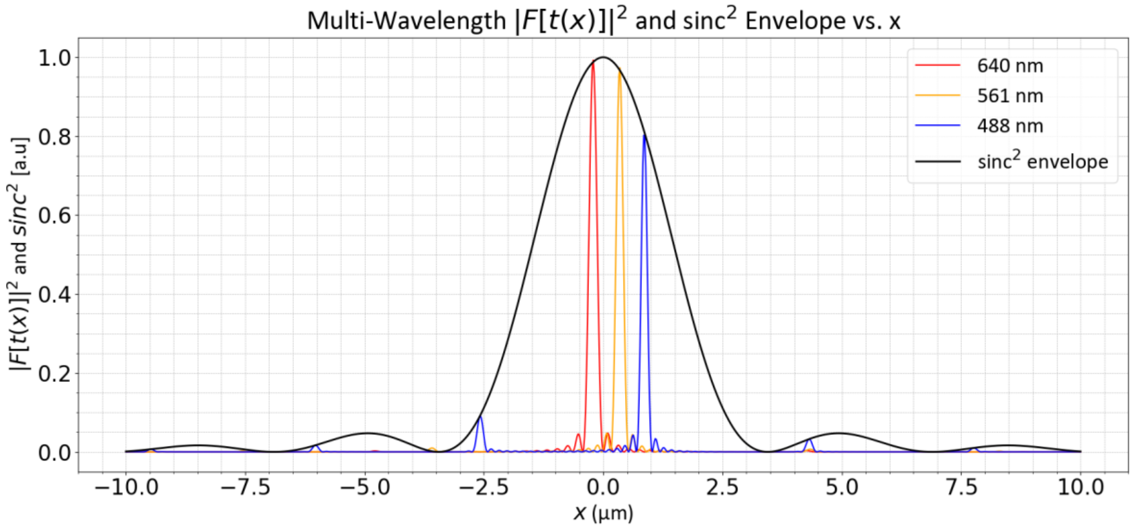

Figure S4 – Single-order Terrace simulation ($\Delta n \approx 0.0061$). Calculated $|F\{t(x)\}|^2$compared with the sinc² envelope for 640 nm (red), 561 nm (yellow) and 488 nm (blue). The red wavelength is near-optimal (~100% theoretical).

4.2. Dual-order ratio

We measured, for each wavelength, the 0th to 1st order lobe intensity ratio produced by the Dual-order Terrace and compared it to simulations. The best agreement is obtained for $\Delta n \approx 0.0034$, where the design target for the NeuroPAL component is 0.0027, which should produce a lobe intensity ratio of 50% for 540 nm, and equal intensities between 0th order lob at 460 nm and 1st order lob at 650 nm. Table S3 summarizes the measured and simulated ratios. The simulated $0^{th}/1^{st}$ ratios for 640, 561 and 488nm are 78.1%, 42.7% and 19.4%, respectively (see Figure S5).

λ	640 [nm]	561 [nm]	488 [nm]
Measured Dual-order ratio [%]	80.0%	39.6%	19.3%
Simulated Dual-order ratio [%] for $\Delta n = 0.0034$	78.1%	42.7%	19.4%

Table S3. Dual-order ratio. Integrated intensity ratio 0th / 1st order for each wavelength. Simulated values correspond to $\Delta n = 0.0034$. Balance ratio – intensity of the resulted PSF of 0th order (numerator) and 1st (denominator) of each wavelength.

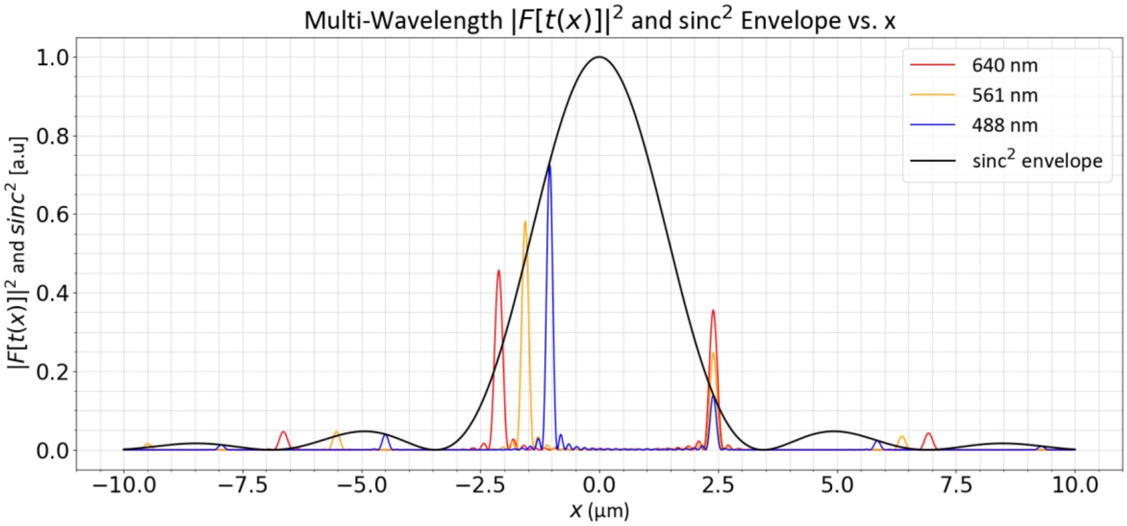

Figure S5. Dual-order Terrace simulation ($\Delta n \approx 0.0034$). Calculated | $F\{t(x)\}$ |² for 640 nm (red), 561 nm (yellow) and488 nm (blue), with the sinc² envelope overlaid. Relative 0th vs. 1st order strengths match the measured ratios.

5. Dichroic mirror Transmission window

In "mRNA barcode imaging" section in the main text, we designed the Single-order Terrace for imaging stretched mRNA molecules labeled with NanoString dyes AF488, Cy3, AF594, and AF647 with emission peaks 520, 570, 617, 670 nm respectively. Although the four channels are separable (as in Fig. 3), discrimination between Cy3 (green) and AF594 (yellow) is intrinsically harder in our setup because they share the same dichroic-mirror transmission window. As shown in Figure S6, the multiband dichroic blocks Cy3 near its 570 nm peak. Consequently, the detected Cy3 emission peak area is blocked and its effective spectrum shifts toward longer wavelengths, while overlapping the AF594 band and reducing color separation.

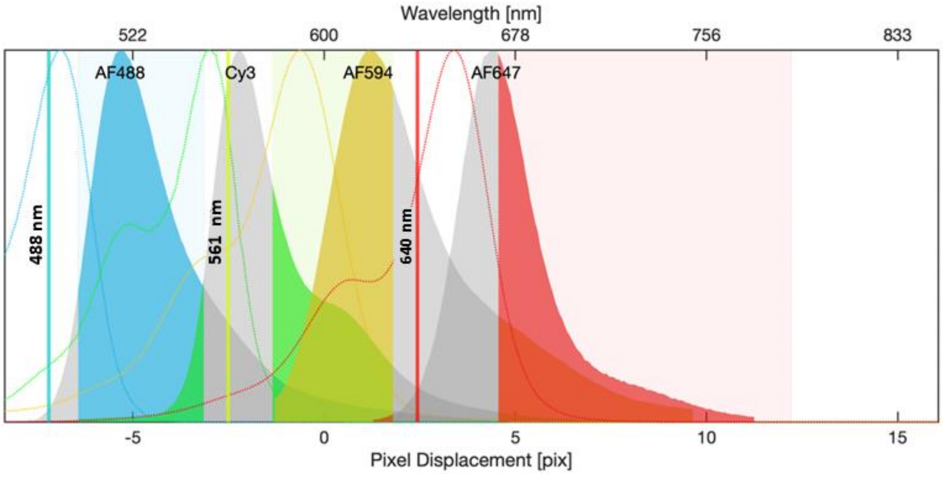

Figure S6 – Normalized excitation (dashed) and emission (solid) spectra of AF488 (cyan), Cy3 (green), AF594 (yellow), and AF647 (red), overlaid with the transmittance window of the Chroma TRF 89902-NK multiband dichroic and the excitation laser lines (vertical markers). Gray regions indicate regions of each emission spectrum blocked by the

dichroic. For Cy3, suppression around 570 nm shifts the detected spectrum toward the AF594 band, which makes Cy3/AF594 separation more challenging. The bottom axis shows pixel displacement (via $d(\lambda)$); the top axis shows wavelength.

6. Spectral Double-helix Ambiguity

The multi-spectral double-helix (Spectral-helix) encodes spectrum and depth in the two lobes of the double-helix PSF: the lobe distance, d, and the lobe angle, θ. Over most of the axial range, the distance angle mapping to spectrum and depth ((d,θ) to (λ, z)) is one-to-one, but there are short regions in (λ, z) parameter space where different wavelength/depth pairs produce nearly identical d & θ pairs. These ambiguous zones are highlighted by dashed ellipses in Figure S7. (a). Even though at most of the range the mapping is unique, some regions, marked in dash ellipse in Figure S7, are ambiguous and represent two depth-spectrum pairs.

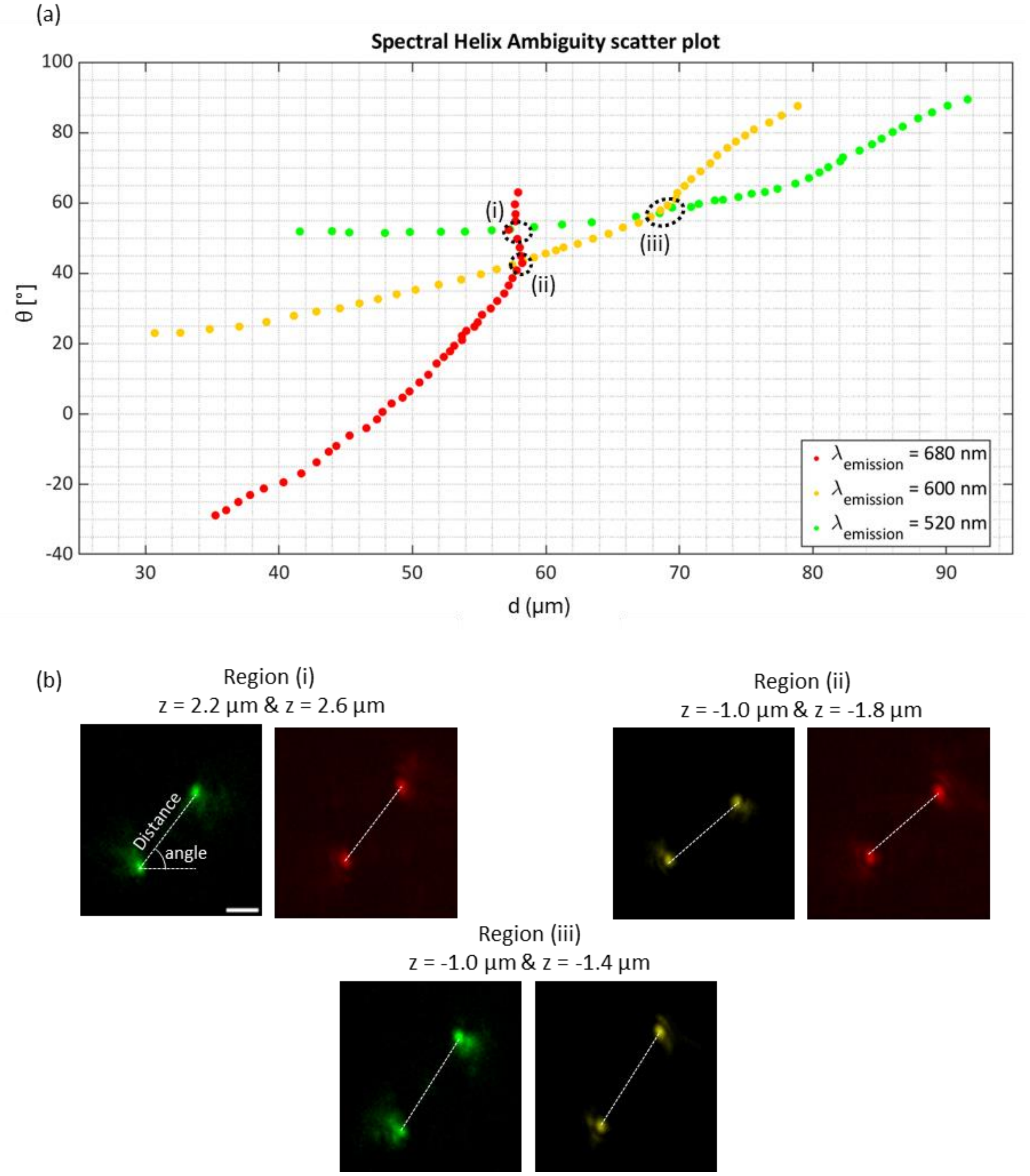

Figure S7. Spectral-helix PSF ambiguity characterization. Measured ambiguity (a) scatter plot: θ (deg) versus inter-lobe distance d(μm) for 520 nm (green), 600 nm (yellow), and 680 nm (red) across the axial range. Dashed ellipses mark regions where distinct (λ, z) pairs yield overlapping (d, θ): (i), (ii) and (iii) as labeled. (b) Example PSFs from each ambiguous region; the white dashed line indicates the measured (d, θ) in each image.

From Figure S7, the ambiguous spots are estimated as: region (i): 600 nm at z ∈ [1.2, 1.8] overlaps with 520 nm at z ∈ [-1.2, -0.8] μm; region (ii): 600 nm at z∈[-0.7, 1.0] μm overlaps with 680 nm at z ∈ [1.6, 2.0] μm; region (iii): 680 nm at z ∈ [2.4, 2.8] μm overlaps with 520 nm at z ∈ [-2.2, -2.1] μm.

Example PSFs from each region are shown in Figure S7. (b). The white line indicates (d, θ) measured for that frame. In practice, the size of the ambiguity regions, marked with ellipses, should be determined by SNR.

7. Field-of-View dependence

During characterization by imaging the four-colored Tetraspeck beads, we observed a lateral chromatic bias that is field-of-view (FOV) dependent, which is orthogonal to the Terrace's designed dispersion axis (vertical). With the Dual-order Terrace inserted behind the 100x/1.35 SI objective, emitters near the horizontal edges of the FOV display a wavelength-dependent horizontal shift relative to the local 0th order. The trend is approximately linear with emitters horizontal positions (Figure S8).

In Figure S8. (a), at the right end of the FOV, the red (~680 nm) replica is shifted slightly to the left and the green (~515 nm) slightly to the right compared to the center 0th order, while the trend is flipped (and weaker) on the opposite end. This also results in coma-like shape on the 0th order. Figure S8. (b) quantifies the effect in object space: over a horizontal span of ±80 μm (the sensor FOV), the Δx bias vs horizontal field coordinate increases monotonically, reaching about 0.35–0.40 μm for 515 nm and about 0.10–0.20 μm for 600/680 nm. The Δy curves vs horizontal field coordinate are essentially flat (≈ constant offsets of ~5, ~4.3 and ~3.7 μm for 515/600/680 nm, respectively), indicating no systematic vertical trend.

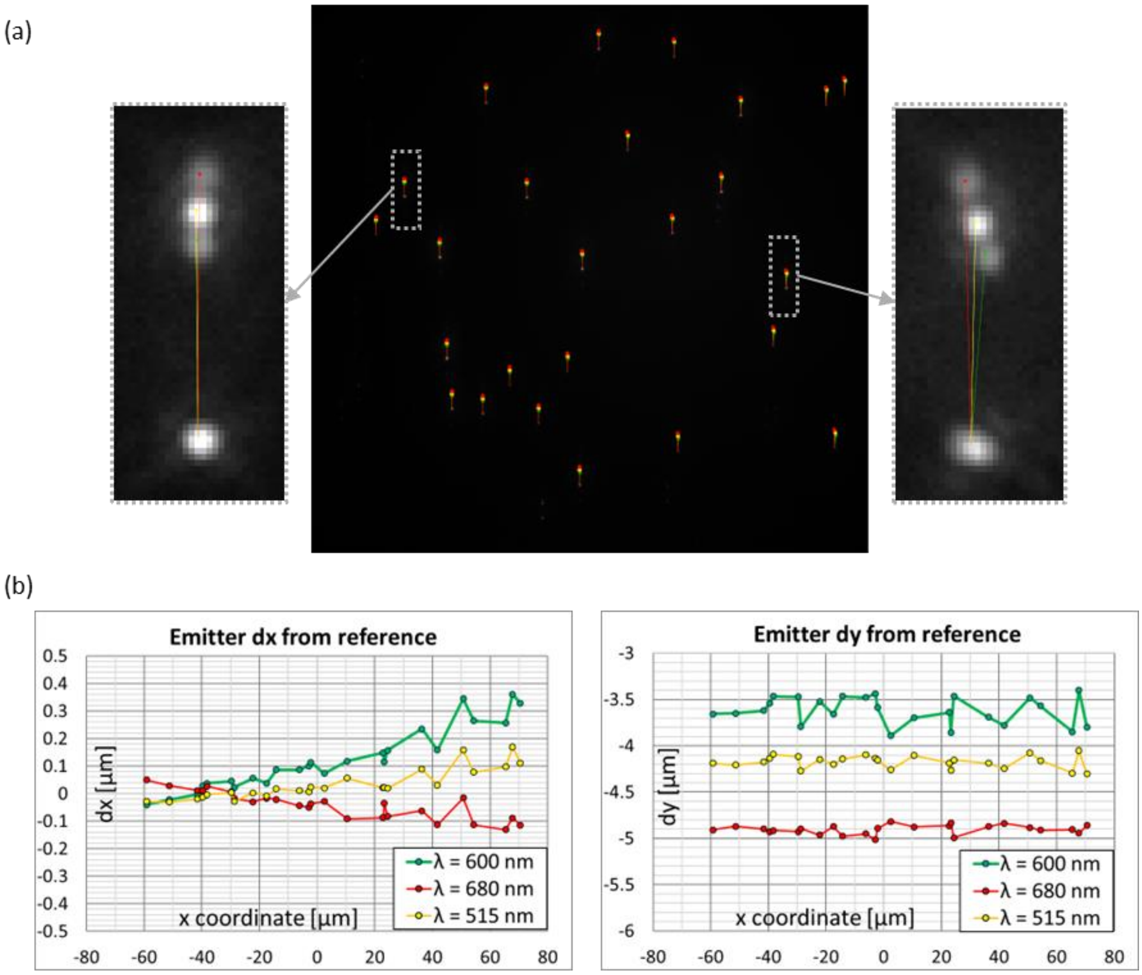

Figure S8. Field-of-view dependence characterization. (a) Full-FOV acquisition with the Dual-order Terrace. Emitters on the right end exhibit a weak horizontal color displacement (red- left, green- right), together with a slight 0th order coma, while emitters on the left end display a weaker effect in the opposite direction. (b) Quantitative analysis in object space. left – horizontal displacement, Δx, of each color relative to the local 0th order vs. horizontal field coordinate x[µm]. The bias increases up to ~0.35–0.40 µm at the far edge for 515 nm. Right – vertical displacement Δy vs. x[µm]. curves are flat, confirming that the dispersion axis (vertical) is not affected by FOV position.

The conclusion of this empiric analysis is that the spectral shift along the dispersion axis is FOV-invariant, whereas a small orthogonal horizontal shear grows with field position. In dense scenes this bias should be considered and handled by per-wavelength FOV calibration with respect to the 0th order in the Dual-order Terrace masks, or with respect to FOV without the mask in the Single-order case.

References

(1) Petrov, P. N.; Shechtman, Y.; Moerner, W. E. Measurement-Based Estimation of Global Pupil Functions in 3D Localization Microscopy. *Opt. Express, OE* **2017**, *25* (7), 7945–7959. https://doi.org/10.1364/OE.25.007945.
